# Online Rumor Diffusion Model Based on Variation and Silence Phenomenon in the Context of COVID-19

**DOI:** 10.3389/fpubh.2021.788475

**Published:** 2022-01-27

**Authors:** Chunhua Ju, Yihao Jiang, Fuguang Bao, Bilin Zou, Chonghuan Xu

**Affiliations:** ^1^Modern Business Research Center, Zhejiang Gongshang University, Hangzhou, China; ^2^School of Management Science and Engineering, Zhejiang Gongshang University, Hangzhou, China; ^3^Academy of Zhejiang Culture Industry Innovation and Development, Zhejiang Gongshang University, Hangzhou, China; ^4^School of Business Administration, Zhejiang Gongshang University, Hangzhou, China

**Keywords:** COVID-19 rumor diffusion, variation, oyster, infection rate, ISVOR model

## Abstract

In the era of mobile internet, information dissemination has made a new leap in speed and in breadth. With the outbreak of the coronavirus disease 2019 (COVID-19), the COVID-19 rumor diffusion that is not limited by time and by space often becomes extremely complex and fickle. It is also normal that a piece of unsubstantiated news about COVID-19 could develop to many versions. We focus on the stagnant role and information variants in the process of rumor diffusion about COVID-19, and through the study of variability and silence in the dissemination, which combines the effects of stagnation phenomenon and information variation on the whole communication system in the circulation of rumors about COVID-19, based on the classic rumor SIR (Susceptible Infected Recovered) model, we introduce a new concept of “variation” and “oyster”. The stability of the new model is analyzed by the mean field equation, and the threshold of COVID-19 rumor propagation is obtained later. According to the results of the simulation experiment, whether in the small world network or in the scale-free network, the increase of the immure and the silent probability of the variation can effectively reduce the speed of rumor diffusion about COVID-19 and is conducive to the dissemination of the truth in the whole population. Studies have also shown that increasing the silence rate of variation can reduce COVID-19 rumor transmission more quickly than the immunization rate. The interesting discovery is that at the same time, a higher rumor infection rate can bring more rumors about COVID-19 but does not always maintain a high number of the variation which could reduce variant tendency of rumors. The more information diffuses in the social group, the more consistent the version and content of the information will be, which proves that the more adequate each individual information is, the slower and less likely rumors about COVID-19 spread. This consequence tells us that the government needs to guide the public to the truth. Announcing the true information publicly could instantly contain the COVID-19 rumor diffusion well rather than making them hidden or voiceless.

## Introduction

Information diffusion is a typical model of dissemination in human society. Whenever an emergency occurs, such as the occurrence of COVID-19, it is easy for rumors about COVID-19 to appear and to swirl due to asymmetric information, fuzzy facts, and subjective conjectures (1), which have numerous negative effects. With the rapid development of mobile networks and various social applications, the speed and breadth of information dissemination have changed qualitatively. This has demanded new requirements for relevant organizations to effectively guide the public opinion about COVID-19 and to reduce the harm of rumors about COVID-19.

The research on the propagation model of rumors started in the 1970s. Daley and Kendall (2) proposed a Daley-Kendall (DK) model that combines rumor diffusion with virus infection and divides the individuals into three groups: people who have not heard of the rumor, people who actively spread it, and people who help stop the rumor. As the follow-up research continued, Maki and Thompson (3), based on the DK model, assumed that when a rumor spreader contacts with another spreader, only the former spreader can be transformed into an immunizer. With the development of modern technology, scholars have started to consider the complexity of large-scale social networks. For the first time, Zanette and Damián (4) put a model of rumor propagation in a small-world network environment and obtained its threshold. Subsequently, Moreno et al. (5) replaced the environment of rumor propagation with a scale-free network, described the propagation process with an average field equation, and verified it with a random analysis and computer simulation. Pan et al. (6) studied the propagation simulation of rumors on a scale-free network with a power law distribution and variable clustering coefficients and found that increasing the variable clustering coefficient can effectively prevent the diffusion of rumors and increase the information transparency which helps to dispel rumors.

Nekovee et al. (7) combined the phenomenon that some people stop spreading on account of forgetting rumors in real life, integrated the forgetting mechanism into the classic SIR rumor model, and analyzed its threshold. On this basis, Zhao et al. (8) improved and carried out simulation experiments with LiveJournal data. It was found that the network average degree, forgetting parameters and immune parameters, had significant effects on the rumor diffusion. Later Zhao et al. (9) added the hibernator role with the forgetting and memory mechanism and proposed a new Susceptible-Infected-Hibernator-Removed (SIHR) model. Wang (10) introduced a trust mechanism and concluded that trust factors can effectively reduce the size of the final rumor diffusion and delay the process, but it will increase the threshold of propagation in the network. Wang et al. (11) divided the people into four categories based on the forgetting mechanism, and proposed SIRaRu. The Ra is used to indicate those who have heard rumors but have no interest in spreading them. The Ru stands for those people who are completely immune to rumors. It has a significant impact that the forgetting parameter can determine the final propagation threshold. Wang et al. (12) proposed a Credulous-Spreader-Rationals (CSR) model based on the increasing convenience of social networks, which divided the nodes in the group into three categories: credulous, spreader, and rational. Huo and Huang (13) applied the idea of system dynamics to integrate the influence of popular science propaganda and media reports on the diffusion of false information, and proposed an optimal control strategy. Wang et al. (14) extended the forgetting mechanism, set the forgetting rate which could be changed over time, and proved that a larger initial forgetting rate or forgetting speed can reduce the size of the final rumor. Zhang et al. (15) constructed a net rumor diffusing model with a social strengthening mechanism and attenuation characteristics and proved that intervention in public interest can effectively curb rumor diffusion. Liu et al. (16) studied the modified Susceptible-Exposed-Infectious-Recovered (SEIR) model on a scale-free network and compared the effectiveness of the two strategies of group immunity and target immunity. Li and Ma (17) constructed a two-tier social network, which is online and offline, to describe the diffusion of public opinion. Seoyong and Sunhee (18) analyzed rumor data collected from the social survey about Fukushima nuclear accident. They think the best way to neutralize rumors is by reducing the perceived risk and negative information, and enlarging the source credibility, perceived benefit, trust, and knowledge. Zhu and Ma (19) proposed a Susceptible-Hesitated-Infected-Removed (SHIR) model with hesitant roles on heterogeneous random networks based on the individual dynamic relationship changes and subjective judgments, and people who expressed individual choices through the weight of edges. Zan (20) proposed a Double-Susceptible-Infectious-Recovered (DSIR) (Double Rumor) model, where two rumors compete with one another. The selection parameters are used to represent the attractiveness of each rumor, while the time delay parameter is used to represent the time difference between each other. Amaral and Arenzon (21) proposed a propagation model with skeptical characters and simulated the symbiosis of survivor zombies to characterize the equilibrium status of each character during the diffusion of rumors. Zhu and Wang (22) established a rumor propagation model with uncertainty based on the spatiotemporal diffusion framework. Experiments proved the uncertainty of network topology and human behavior when it reaches to the threshold and determines the density of infected connections. With the improvement of specific capabilities of society, rumors diffuse to a stable state at a faster rate. Ankur et al. (23) found that the different interests of each person are uncertain and volatile about rumor propagation. Therefore, a deterministic model and a random model were established on a homogeneous network, and a deterministic equation was introduced on the random uniform network to discuss if the noise could extend the diffusion of rumors. Liu and He (24) proposed a non-linear dynamic propagation model for information competition from the viewpoint of public opinion control. The Markov chain theory was used to analyze the node state transition matrix in the perspective of competition and verified the feasibility of competing information dissemination with the empirical analysis of hot events, which was used for comparison. Zhang et al. (25) added a communicator who will diffuse real information, combined with the effect of forgetting mechanism on propagation, and used the regeneration matrix to obtain the threshold for simulation experiments. Guo et al. (26) designed a multi-feature diffusion model (MF-model), formulated a multi-feature rumor blocking (MFRB) problem on a multi-layer network structure, and proposed a Revised-IMM algorithm, considering the spread of rumor is determined by multiple features. Leonid (27) modified the spreader which is divided into the low rate of active spreaders and the high rate of active spreaders, and got its stability under stochastic perturbation with the method of linear matrix inequalities. Guo et al. (28) proposed an overall evaluation on benefits of influence (OEBI) problem, based on the phenomenon that a user is influenced by both our own information and the information of our rival. They proved the objective function of the OEBI problem is not monotone, not submodular, and not super modular.

However, with the continuous deepening of research on rumors, the temporary non-spreading role in the middle state also needs to be considered in the rumor propagation model, which has played a temporary buffer role in the diffusion. At the same time, the instability of the turbulent information has a strong offset property, which causes the variation of rumors in various versions. In the uninterrupted diffusion of rumors, there is a lot of chaos based on subjective emotions derived from objective factors. During the diffusion of rumors, with various mutations and distortions, there will be numerous new descriptions that are far different from the original version. Therefore, mutational rumors display different effects on rumor propagation.

In the context of the COVID-19 outbreak, and based on the classic rumor SIR model, this research combines the effects of stagnant roles and information variants in the circulation of COVID-19 rumors on the entire communication system, introduces new concepts of mutants and silent people, and proposes the ISVOR (Ignorance-Spreader-Variation-Oyster-Recovery) model. The research also calculated the corresponding average field equation, and further studied its propagation law and the influence of various parameters during the propagation. The research work has profoundly explored the theory of new rumor propagation model, and it has practical significance for the government or enterprises to effectively guide forward the public opinion of COVID-19 on the Internet.

The main contributions are summarized as follows:

- In order to accurately study the propagation of rumors during COVID-19, two novel variables called “variation” and “oyster” are introduced. Then an ISVOR model with Ignorance-Spreaders-Oyster is proposed.
- In order to find the balanced state of the rumor spreading quickly, we use the average field equation to derive the threshold value of opinion propagation.
- The findings can better help the government and relevant departments in responding to rumor control in emergencies.

## Problem Analysis

**Figure 1:**
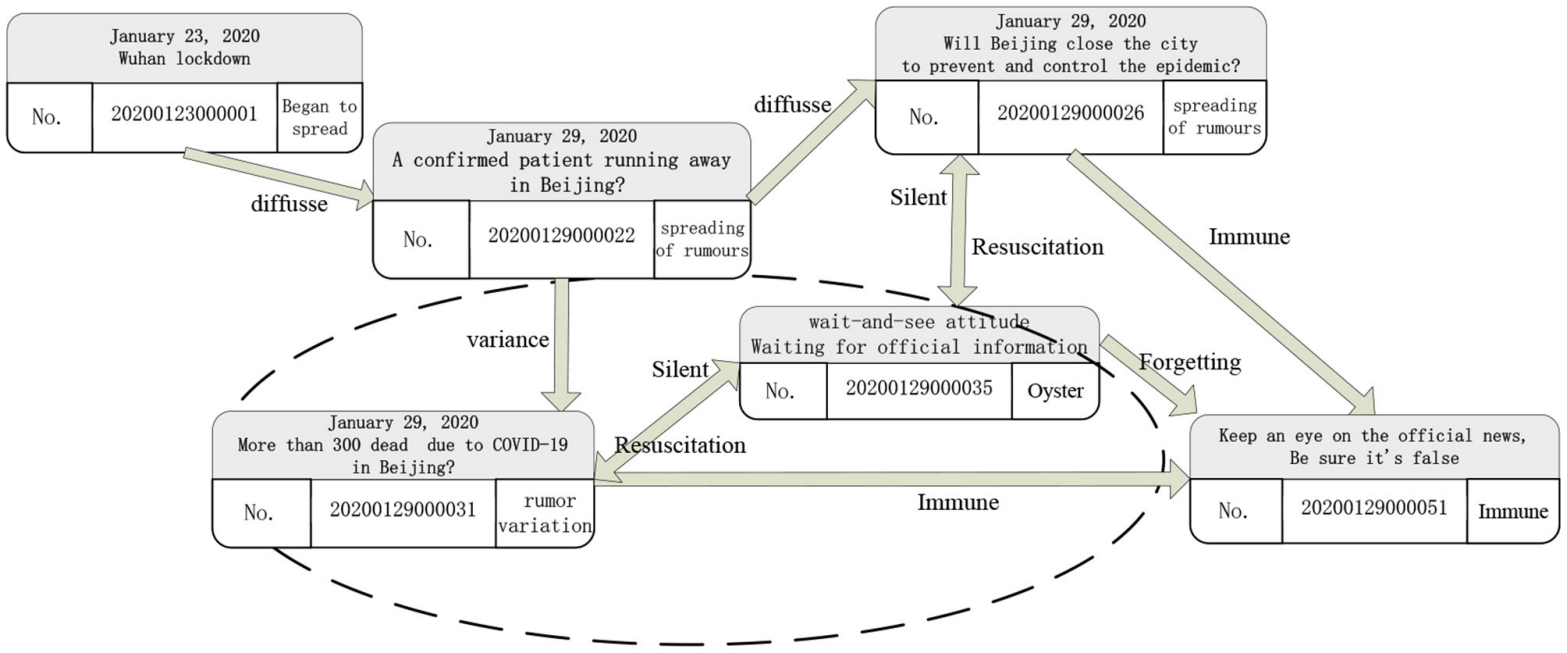
The spread of “new crown epidemic” rumors (number is timestamp).

The COVID-19 is a global health emergency that is having a profound impact on the physical and mental health of people. Take the information “Wuhan lockdown”, for example, that once diffused online. During COVID-19, some widely circulated but unconfirmed news on social media, such as that the salt brine mouthwash and smoked vinegar could prevent viruses, and some people heard the news and went to grab these items, causing them to sell out, while those who did not grab the items felt the social inequality caused by the information asymmetry, but these unconfirmed messages were eventually confirmed as rumors by public media and experts. A study shows that, in the context of the increasingly sound development of new media functions, the elderly population is enjoying short videos spread through WeChat and Microblog, and the authenticity of which cannot be verified. These contents were often identified as rumors (12). Among those people who heard this news, some people were reflecting on the news or were waiting for the news from the official website which could be in a silent state of not spreading. After a period of silence, some silencers became immune to this rumor because of rational thinking or other reasons. Some silencers might return to the state of “diffusing rumors”. The rumor will continue to mutate under the turmoil. Previous studies have often ignored the role of “silent people” and focused on the own propagation of the communicator. Also, the rumor variation in the process and the influence of variation instability factors on the entire communication chain were neglected, as circled in Figure 1.

The typical instability of rumors makes it easily but incorrectly relayed. Especially in the event of sudden events to satisfy the curiosity of the public about unknown things, a variety of information, which is hard to detect, will be circulated in society, particularly on the Internet. Although the information itself is full of various uncertain factors, according to their own needs and psychological reasons (1), many people will involuntarily believe the rumors, then substitute their own subjective assumptions, amplify the empathy of the group, and continue to promote the development of rumors. In the process of online communication, frictions can occur among individuals. The trajectory of the rumors will be deflected and twisted, and the rumors will begin to mutate. At the same time, calm and rational attitudes are mixed with blind following. Disputes of different views come on stage in turn.

## Improved ISVOR Model Based on SIR Model

This paper is based on the SIR epidemic model, the ISVOR model was constructed by introducing two novel variables of “variation” and “oyster”.

### SIR Model

In the 1860s, Daley and Kendal found the similarities between infectious diseases and information transmission by comparing them. They first proposed the classic DK model (2), that is, the SIR model, which is the most widely used. In this model, the population is abstractly divided into three categories: susceptible, infected, and recovered individuals, corresponding to the individuals who do not know the information, the individuals who transmit the information and the individuals who no longer participate in the information transmission.

### ISVOR Model

The ISOVR model was established based on the classical SIR rumor propagation model by introducing the novel role concepts of mutators and silencers, and the concepts of stagnant roles and information variants were proposed, which were defined as follows:

#### Stagnant Role

When certain nodes in the network, i.e., real people, choose to remain in a brief state of stagnation by not spreading rumors after receiving them. However, this role will change again as the rumor continues to spread, and finally remain immune to the rumor or return to the state of rumor spreading.

#### Information Variants

As rumors spread on the Internet, they are altered by some people, amplifying the uncertain content of the information, and adding their own distorted understanding or deliberate misinterpretation of the fabrication. The mutated rumors change the speed of their dissemination, thus, causing other actors to be affected in various ways in the process of rumor spreading.

In the following, we will define the variables of the ISVOR model:

#### Ignorance

People who have not yet heard rumors about COVID-19 and could easily believe the rumors about COVID-19.

#### Spreader

Ordinary rumor spreader, people who contacted the earliest version of the rumors about COVID-19 and believed it. Also, they would spread the rumors about COVID-19 actively.

#### Variation

Variable spreader, which is different from the ordinary spreader. The content or version of rumors about COVID-19 that the variable spreader carries has transformed a lot than before, the property of the message and specific information have changed, and there is a trend of continuous change.

#### Oyster

People who received the information about COVID-19 rumors, but stay silent, will not actively spread it, in a state of thinking or bystander.

#### Recovery

People who know the truth of the rumors about COVID-19 and refuse to spread the rumors about COVID-19, and can help others enter the immune state.

#### Forgetting

After a period of continuous silent thinking or being bystander, the oyster forgets rumors about COVID-19 or ignores rumors about COVID-19 and transforms into the recovery.

#### Resuscitation

After a period of continuous silent thinking or being bystander, the oyster re-enters the state of rumors about COVID-19 spreading or transforms into the variation.

#### Immune

Understanding the truth or refuse to spread the rumors about COVID-19, and owning the ability to help others transform into the recovery.

**Figure 2:**
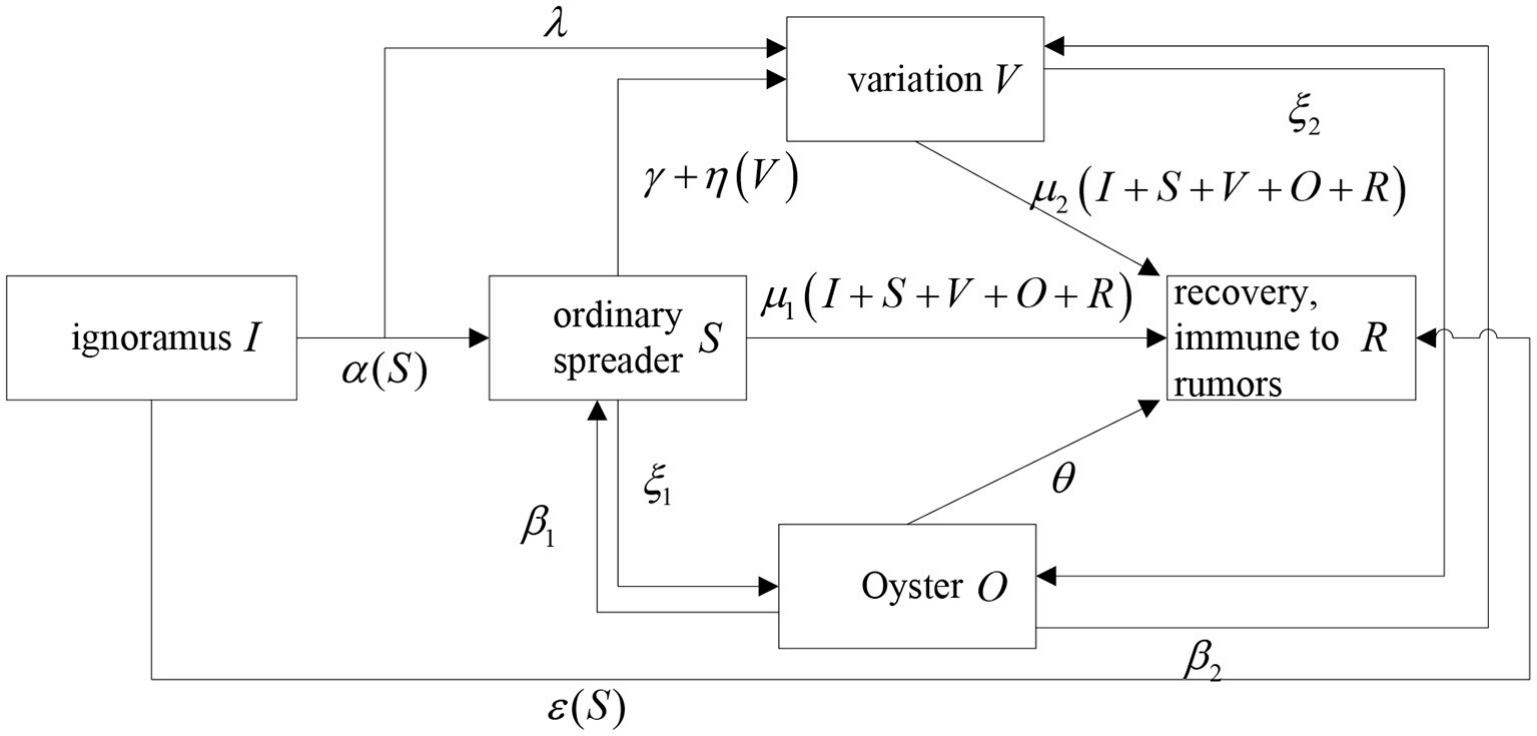
Model structure diagram.

Assuming there is an evenly mixed social network with *N*vertices, vertices represent individuals in the society, while the edges represent the connection between two individuals. In this way, an undirected graph can be obtained, which is a set of all vertices and a set of all edges. The diffusion of rumors about COVID-19 can be abstracted into the model shown in Figure 2.

The symbols are explained as follows:

*I*: ignoramus, in the state of ignorance in the information network, is easily susceptible to rumors about COVID-19 ; *S*: ordinary spreader, prophase rumors about COVID-19 carrier, believes and diffuses rumors about COVID-19 and can infect the ignorance; *O*: oyster, who do not spread rumors about COVID-19 due to factors such as reflection on rumors; *R*: recovery, immune to rumors about COVID-19, who does not spread rumors about COVID-19 and even spread the truth ; *V*: variation, a special spreader, whose content or property mutated from some early versions comparing with ordinary spreader's rumors about COVID-19.

α: the probability of the ignorance infected by rumors about COVID-19 to become the spreader; ε: the probability of ignorance contacted with rumors about COVID-19 to become the recovery; λ: the probability of ignorance to become the variation among those who are infected.

μ_1_: the immunity probability of the spreader that becomes the recovery under the influence of other roles; γ: the probability of the spreader that spontaneously becomes the oyster; ξ_1_: the silent probability of the spreader that becomes the oyster; η: the probability of the spreader coming into contact with the variation and becoming the variation.

μ_2_: the immunity probability of the variation becoming the recovery under the influence of other roles; ξ_2_: the silent probability of the variation that becomes the oyster.

θ: the probability of the oyster to forget the rumors; β_1_: the probability of the oyster that goes back into the spreader; β_2_: the probability of the oyster that goes back into the variation.

The process of rumors about COVID-19 diffusion is as follows.

- When in contact with the spreader, the ignorance with the probability α turns into the spreader. Among them, some is transformed into the variation with the probability λ;
- When in contact with the spreader, the ignorant is transformed into the recovery with the probability ε;
- When in contact with other roles like the spreader, variation, oyster, and recovery, the spreader turns into the recovery with the probability μ_1_;
- When there is no contact with the variation, the spreader turns into the variation with the probability γ; when in contact with the variation, it turns into the variation with the probability η;
- When in contact with others, the variation turns into the recovery with the probability μ_2_;
- The spreader and the variation become the oyster with probability ξ_1_ and ξ_2_. The oyster may forget the rumors about COVID-19 or lose interest in it then no longer spread it which becomes the recovery with the probability θ;
- The oyster will be resuscitated as the spreader and the variation, again, with the probability of β_1_ and β_2_.

The model divides all the people in the whole group into five roles. Each role has its own specific probability, which is conducive to make a concrete analysis of the situation after the COVID-19 rumor occurs in emergency circumstances.

## Steady State Analysis of COVID-19 Rumor Propagation

Mean field theory is to average the applied force exerted by the surrounding environment of objects (29), quantifying the information of a physical model, which is widely used in complex systems and electromagnetics. The mean field theory replaces the effect of the environment on objects with the average effect instead of the individual effects and can average the influence of other nodes in the complex network on itself.

In this model, *I*(*t*), *S*(*t*), *V*(*t*), *O*(*t*), and *R*(*t*) each represents the density of the ignorance, the spreader, the variation, the oyster, and the recovery in the network at time *t*. We can get the average field equation of the ISVOR model in the network:


$$\begin{array}{l} \{\begin{array}{c} \frac{dI(t)}{dt}=-k\alpha I(t)S(t)-k\lambda I(t)V(t)\\ \frac{dS(t)}{dt}=-\mu 1kS(t)(S(t)+V(t)+O(t)+R(t))-(\xi 1+\gamma )S(t)\\ +\alpha kI(t)S(t)+\beta 1Ok(t)-\eta kS(t)V(t)\\ \frac{dV(t)}{dt}=-\mu 2kVk(t)(S(t)+V(t)+O(t)+R(t))-\xi 2V(t)\\ +\gamma S(t)+k\lambda I(t)V(t)+\eta kS(t)V(t)+\beta 2O(t)\\ \frac{dO(t)}{dt}=-(\beta 1+\beta 2)O(t)+\xi 1S(t)+\xi 2V(t)\\ -\theta O(t)(S(t)+V(t)+O(t)+R(t))\\ \frac{dR(t)}{dt}=\theta (t)O(t)+\mu 1kS(t)(S(t)+V(t)+O(t)+R(t))\\ +\mu 2kV(t)(S(t)+V(t)+O(t)+R(t)) \end{array} \end{array}$$

Setting the left part of the equation to 0, we can formulate the non-negative equilibrium solution *E*_1_ = (*I*_1_, 0, 0, 0, 1 − *I*_1_), *E*_2_ = (0, *S*_2_, *V*_2_, *O*_2_, 1 − *S*_2_ − *V*_2_ − *O*_2_), *E*_3_ = (*I*_3_, *S*_3_, *V*_3_, *O*_3_, 1 − *S*_3_ − *V*_3_ − *O*_3_ − *I*_3_). Among those, for *E*_1_, when *I*_1_ equals any non-negative real number <1, these equations hold obviously.

For *E*_2_, given that $\{\begin{array}{c} \begin{array}{c} S_{2}>0\\ V_{2}>0 \end{array}\\ O_{2}>0\\ 1-S_{2}-V_{2}-O_{2}>0 \end{array}\;$, we can obtain the equation that μ_1_*kS*_1_ + μ_2_*kS*_2_ + θ*O*_2_ = 0, which is not correspond with the fact. Thus, there is no variable for *E*_2_.

For *E*_3_, given that $\{\begin{array}{c} \begin{array}{c} I_{3}>0\\ S_{3}>0 \end{array}\\ V_{3}>0\\ O_{3}>0\\ 1-S_{3}-V_{3}-O_{3}>0 \end{array}\;$, we can obtain the equation that α*k*_*I*_3_*S*3_ + −λ*k*_*I*_3_*V*3_ = 0, which is not correspond with the fact either. Thus, there is no variable for *E*_3_.

To summarize, there is only one equilibrium solution *E*_1_ = (*I*_1_, 0, 0, 0, 1 − *I*_1_) (*I*_1_ > 0).

Referring to Li's mathematical method (30), assuming E^*^ = (I^*^, S^*^, V^*^,O^*^,R^*^) is the solution, we can get the following:


$$\begin{array}{l} \left\{ \begin{array}{c} \frac{dY(t)}{dt}=(\alpha kS^{*}+\mu 1kS*)X(t)+(\mu 1kI^{*}-\mu 1k-\xi 1-\gamma +\alpha kI^{*}-\eta kV^{*})Y(t)-\\ \eta kS^{*}Z(t)+\beta 1W(t)+(\mu 1k+\alpha k)X(t)Y(t)-\eta kY(t)Z(t)\\ \frac{dZ(t)}{dt}=(\lambda kV^{*}+\mu 2kV^{*})X(t)+(\gamma +\eta kV^{*})Y(t)+\\ (\mu 2kI^{*}-\mu 2k-\xi 2+k\lambda I^{*}+\eta kS^{*})Z(t)+\\ \beta 2W(t)+(\mu 2k+\lambda k)X(t)Z(t)+\eta kY(t)Z(t)\\ \frac{dW(t)}{dt}=\xi 1Y(t)+\xi 2Z(t)-(\beta 1+\beta 2+\theta )W(t) \end{array}\right. \end{array}$$

We substitute the variable, then can get the Jacobian matrix of coefficient.


$$\begin{array}{l} \begin{array}{l} \begin{array}{l} J(E^{\prime })=\left(\begin{array}{ccc} \mu 1kI_{1}-\mu 1k-\xi 1-\gamma +\alpha kI_{1} & 0 & \beta 1\\ \gamma & \mu 2kI_{1}-\mu 2k-\xi 2+\lambda kI_{1} & \beta 2\\ \xi 1 & \xi 2 & \theta I^{*}-(\beta 1+\beta 2)+\theta X(t) \end{array}\right) \end{array} \end{array} \end{array}$$

Supposing two of the characteristic values χ_1_ = *U*_1_, χ_2_ = *U*_2_, then $tr\left(J\left(E^{\prime }\right)\right)=U_{1}+U_{2}+\theta I_{1}-\left(\beta_{1}+\beta_{2}\right)$. We can obtain χ_3_ = θ*I*_1_ − (β_1_ + β_2_) and the characteristic equation equals 0.


$$\begin{array}{l} \chi^{3}-\left(U_{1}+U_{2}+B\right)\chi^{2}+\left[\left(U_{1}+U_{2}\right)B+U_{1}U_{2}\right]\chi -U_{1}U_{2}B=0 \end{array}$$

where *U*_1_ = μ_1_*kI*_1_ − μ_1_*k* − ξ_1_ − γ + α*kI*_1_, *U*_2_ = μ_2_*kI*_2_ − μ_2_*k* − ξ_2_ + λ*kI*_1_, *B* = θ*I*_1_ − (β_1_ + β_2_).

According to stability theory and Hurwitz theorem, if the system is in stable, we can have Δ_1_ = −(*U*_1_ + *U*_2_ + *B*) > 0, Δ_2_ = −(*U*_1_ + *U*_2_ + *B*)[(*U*_1_ + *U*_2_)*B* + *U*_1_*U*_2_] − *U*_1_*U*_2_*B* > 0, Δ_3_ = −*U*_1_*U*_2_*B* > 0. Additionally, we can get that $\{\begin{array}{c} U_{1}<0\\ U_{2}>0\\ B>0 \end{array}\;$ or $\{\begin{array}{c} U_{1}>0\\ U_{2}<0\\ B>0 \end{array}\;$. Thus, if the system is in stable, *I*_1_ needs to satisfy the $\{\begin{array}{c} \frac{\mu_{1}k+\xi_{1}+\gamma }{\mu_{1}k+\alpha k}<I_{1}<\min\left(\frac{\mu_{2}k+\xi_{1}+\gamma }{\mu_{2}k+\lambda k},\frac{\beta_{1}+\beta_{2}}{\theta }\right)\;\left(\frac{\mu_{1}k+\xi_{1}+\gamma }{\mu_{1}k+\alpha k}<\frac{\mu_{2}k+\xi_{1}+\gamma }{\mu_{2}k+\lambda k}\right)\\ \frac{\mu_{2}k+\xi_{1}+\gamma }{\mu_{2}k+\lambda k}<I_{1}<\min\left(\frac{\mu_{1}k+\xi_{1}+\gamma }{\mu_{1}k+\alpha k},\frac{\beta_{1}+\beta_{2}}{\theta }\right)\;\left(\frac{\mu_{1}k+\xi_{1}+\gamma }{\mu_{1}k+\alpha k}>\frac{\mu_{2}k+\xi_{1}+\gamma }{\mu_{2}k+\lambda k}\right) \end{array}$.

To sum up, when $\{\begin{array}{c} \frac{\mu_{1}k+\xi_{1}+\gamma }{\mu_{1}k+\alpha k}<I_{1}<\min\left(\frac{\mu_{2}k+\xi_{1}+\gamma }{\mu_{2}k+\lambda k},\frac{\beta_{1}+\beta_{2}}{\theta }\right)\;\left(\;\frac{\mu_{1}k+\xi_{1}+\gamma }{\mu_{1}k+\alpha k}<\frac{\mu_{2}k+\xi_{1}+\gamma }{\mu_{2}k+\lambda k}\right)\\ \frac{\mu_{2}k+\xi_{1}+\gamma }{\mu_{2}k+\lambda k}<I_{1}<\min\left(\frac{\mu_{1}k+\xi_{1}+\gamma }{\mu_{1}k+\alpha k},\frac{\beta_{1}+\beta_{2}}{\theta }\right)\;\left(\frac{\mu_{1}k+\xi_{1}+\gamma }{\mu_{1}k+\alpha k}>\frac{\mu_{2}k+\xi_{1}+\gamma }{\mu_{2}k+\lambda k}\right) \end{array}$, the equilibrium solution *E*_1_ = (*I*_1_, 0, 0, 0, 1 − *I*_1_) exists and there is a global asymptotic stability of the system. Regarding the spreading process of COVID-19 rumors shown in Figure 2, as time increases, eventually all the Ignorance, the Spreader, the Variation, the Oyster, and the Recovery will tend to stop and no longer disseminate any rumors, and, finally, the Ignorance in the system may stabilize at a certain constant value. In real life, for any propagating rumors during COVID-19, once any rumors ferment, it will have a major impact on social harmony and on the stability of lives of people. For government departments, since the beginning of the spread of rumors, they hope to effectively control all the false information and to better prevent it before it happens. In the next part of this article, we will explore the influence of various parameters of the ISVOR model on the propagation of rumors by means of numerical simulation experiments.

## Simulation Experiment

In this section, we use the Monte Carlo method to simulate the simulation on the Matlab platform to verify the proposed model. For the real complex network environment in the diffusion of rumors about COVID-19, we choose a WS small world network as the representative of uniform network, BA scale-free network as the representative of non-uniform network, the nodes in the network represents the real individuals, and the edge represents the connection between individuals in the real network. We set the total number of individuals *N* = 2000. For the WS small world network, the probability of random reconnection *p* = 0.4, average degree 〈*k*〉 = 10; for the BA scale-free network, average degree 〈*k*〉 = 10 and the power law distribution *P*(*k*) = 2*m*^2^*k*^−3^ where *m* = 5.

### The Changes of Roles During the Diffusion of Rumors About COVID-19

**Figure 3:**
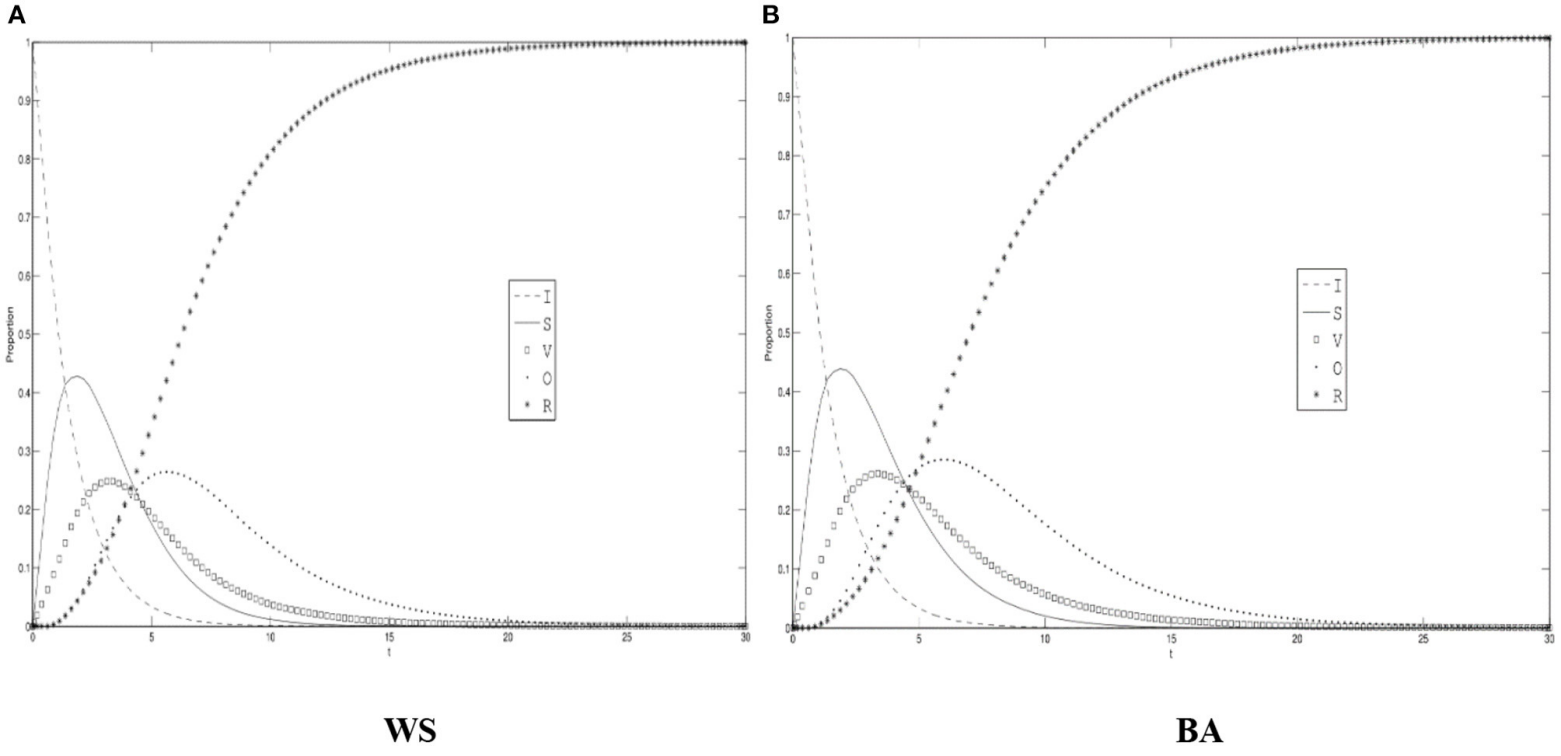
The differences of role density in WS small world and BA scale-free network. **(A)** WS, **(B)** BA.

Figure 3 shows the density changes of the ignorant, the spreader, the variation, the oyster, and the recovery under the WS small world and BA scale-free networks with the default parameters. As can be seen from Figure 3, during the course, the number of the spreader, the variation, the oyster has experienced rapid growth and then steadily reached the peak after a rapid decline process, the number of the ignorant quickly reduces at the beginning and gradually goes down to zero, the recovery rises rapidly and then stabilizes to the highest value. Due to the character of the individual, the spreader appeared before the variation and the oyster. When the rumors about COVID-19 begins to diffuse, the spreader comes to the stage in large numbers. During the individual-to-individual interaction, the information about COVID-19 rumors that carried starts to change, which led to the emergence and swift increase of the variation. When the spreader reaches the threshold, its number goes into a declining period, and people who propagate the rumor in the network will be composed of the spreader and the variation. Due to the factors such as individual thinking and environment, some of the spreader and the variation will temporarily stop diffusing, and a large number of oysters also arise at the very moment, and will occupy a large proportion.

### Comparison Between SIR and ISVOR Models

**Figure 4:**
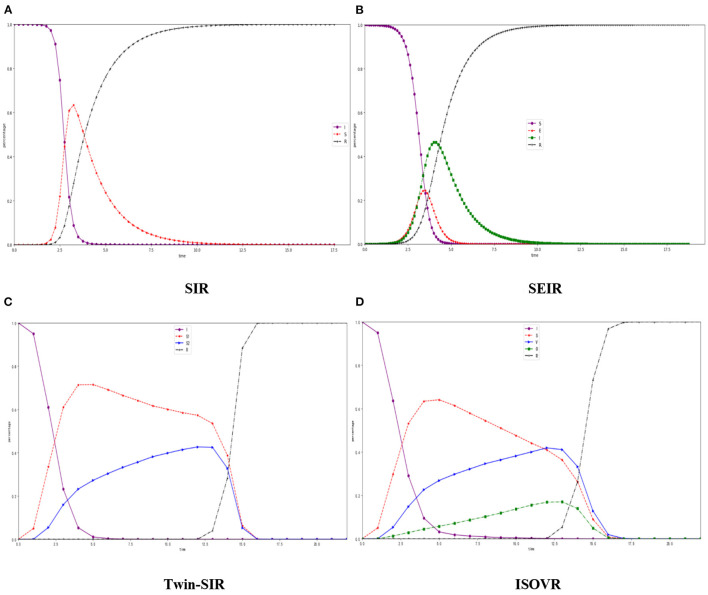
Susceptible Infected Recovered (SIR) model, SEIR model, Twin-SIR, model and ISOVR model in the BA net scale-free network**. (A)** SIR, **(B)** SEIR, **(C)** Twin-SIR, **(D)** ISOVR.

The classic rumor models, SIR, SEIR (31), Twin-SIR (32), and our ISVOR, are used to analyze the spreading process of rumors about COVID-19. In the model SIR, *S* is considered as “susceptible” (people who have not been exposed to rumors about COVID-19), *I* is considered as “infective” (people who are infected with rumors about COVID-19 and can spread), *R* is considered as “remover” (people who are immured with rumors about COVID-19). In the SIR model, we choose α as the probability that *S* accepts rumors about COVID-19 and becomes *I*, and choose μ_1_ as the probability that *I* changes into *R*. Figure 4 shows the density changes of the SIR, SEIR, twin-SIR, and ISOVR models in the scale-free network when the total number of nodes is *N* = 2000, α = 0.5, β_1_ = 0.1, β_2_ = 0.1, λ = 0.2, η = 0.5, ξ_1_ = 0.5, ξ_2_ = 0.5, γ = 0.3, θ = 0.25, μ_1_ = 0.5, and μ_2_ = 0.5.

The classical SIR model has only a simple relationship from *S* to *I* or *I* to *R*. As can be seen in Figure 4A, the number of *S* decreases rapidly, and *I* continues to increase until it decreases after the peak. During this period, *R* starts to appear and continues to increase. We use the number of *S* and *V* to represent the people who diffuse rumors about COVID-19 in the network.

From the curves of the ISOVR model in Figure 4B, the numbers rise faster than in the SIR model, and the peak value is smaller. This is because there is variation in ISVOR. After some of the spreader become the variation which has greater infectivity, thus, accelerating the diffusion of rumors about COVID-19 in the network. At the same time, an “oyster” phenomenon occurs in the group because some people are good at thinking or there are other factors. Some people who diffuse the rumors about COVID-19 will become buffer silencers and reduce the peak of rumor. The principle of “oyster” and “variation” in ISVOR is more complex and reasonable than the simple straight-line relationship in SIR. It is closer to reality which is more in line with the situation of “incorrectly relay an erroneous information” and “ unsuspecting onlookers “ in life.

The SEIR model introduces a hesitating mechanism, which takes the attractiveness and fuzziness of rumor into consideration. The Susceptible (*S*) refers to people who have not been infected with the disease and are, so far, healthy. The Exposed (*E*) is the population that is in the incubation period of this infectious disease, the Infected (*I*) is the population that has been diagnosed, and the Removed (*R*) is the population who has recovered from the rumor. As the rumor spreads from Figure 4B, *E* rises rapidly and will reach the peak firstly, and then, when most of *E* become infected, the Infected will reach an inflection point of growth. With external treatment and autoimmunity, the number of the *E* and infected people will decrease while the number of *R* increased. The Twin-SIR model introduces a new kind of node named “rumor dispeller” with the spreading ability. The rumor dispeller is also in the process of spreading rumor. *The S*_1_ is the spreader, while the *S*_2_ is the rumor dispeller. It can be seen from the Figure 4C that *S*_1_ appears when the rumors begin to spread. With the appearance of *S*_2_, the growth of *S*_1_ is suppressed and the growth rate decreases. The final propagation range, that is, maximum is smaller than SIR and SEIR.

Since ISVOR has a mutation phenomenon, after some of the *S* became *V*, which has greater infectivity, thus, accelerating the diffusion of rumors about COVID-19 in the network. At the same time, an “oyster” phenomenon occurs in the group because some people are good at thinking or due to some especial factors. Some people who diffuse the rumors about COVID-19 will become buffer silencers and reduce the peak of rumor. The ignorant people who affected the surrounding infected more nodes and kept rising at a higher rate. After the nodes collide and interact with each other, more and more *S* turn into *V*. *The V* has a more powerful infectious ability and influence effect, prompting other characters to change in the direction of variation, making the communicators start to decline after reaching the peak. At the same time, due to the individual's own immune ability, some nodes will be in a silent state, neither infected nor spreading, and becomes *O*. After continuous interaction, a part of the *S* and *V* enters the silent interval and turns into *O* with the change of state. The proportion of the *O* keeps a certain proportion and slowly increases until it peaks. When the truth of the rumors begins to spread, those who are immune will quickly spread across the Internet and quickly affected the transformation of the carriers of the rumors. The principle of “oyster” and “variation” in ISVOR is more complex and reasonable than the simple straight-line relationship in SIR. It is closer to reality which is more in line with the situation of “incorrectly relay an erroneous information” and “ unsuspecting onlookers “ in life.

### The Impact of the Variation's Immune Probability on COVID-19 Rumor Diffusion

Assuming max{*S*(*t*) + *V*(*t*)} is the maximum value of the density of the diffuser (which is composed of the spreader and the variation) during the propagation process, that is used to express the maximum contagion capacity of COVID-19 rumors diffusion in the network.

**Figure 5:**
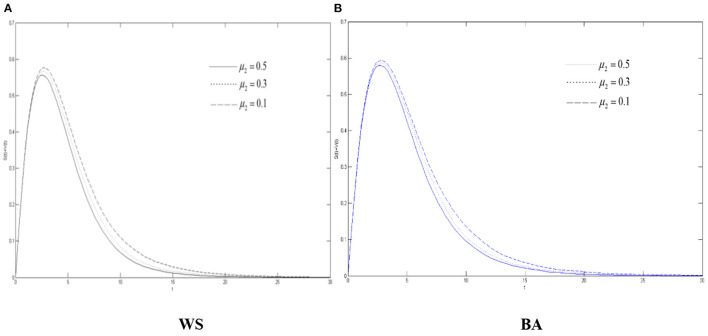
The differences of role density in WS small world and BA scale-free network when μ_2_ changes. **(A)** WS, **(B)** BA.

**Figure 6:**
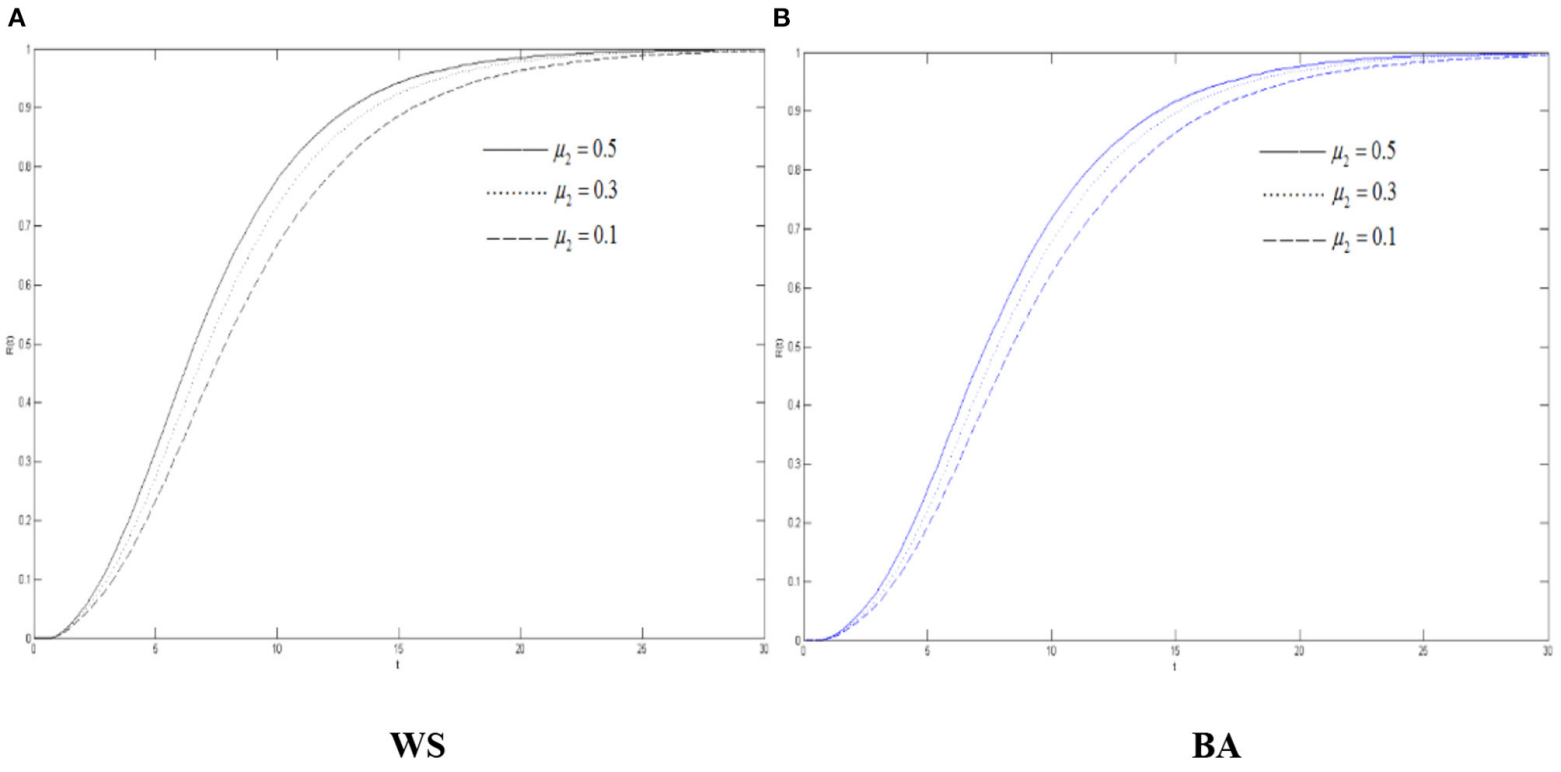
The differences of the recovery density in WS small world and BA scale-free network when μ_2_ changes. **(A)** WS, **(B)** BA.

**Figure 7:**
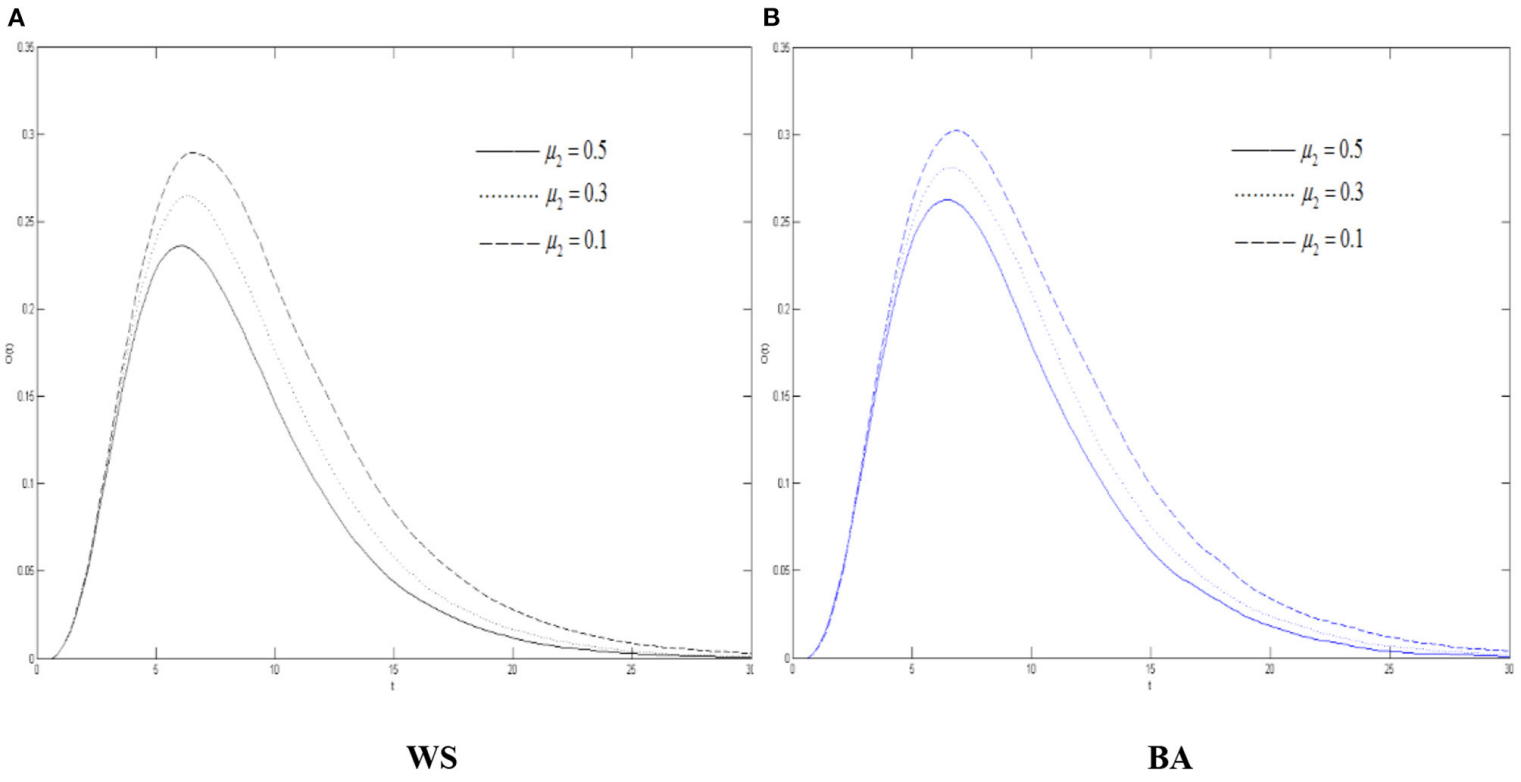
The differences of the oyster density in WS small world and BA scale-free network when μ_2_ changes. **(A)** WS, **(B)** BA.

Figures 5–**7** show the density of the diffuser, the recovery, and the oyster, respectively, in the WS small world network and BA scale-free network when the variation's immune probability μ_2_ will be taken as 0.1, 0.3, and 0.5, respectively. It can be seen from Figure 5 that max{*S*(*t*) + *V*(*t*)} has gone through a process of continuous growth and declining after reaching the peak. Also, it decreases with the increase of the variation's immune probability. When the variation has a lower immunity probability than the spreader, compared with the spreader, more variation is more likely to be in the state of diffusing rumors about COVID-19.

We can also see from Figure 5 that due to the characteristics of the BA scale-free network, the network structure is relatively “fragile” compared to the WS small world. When a hub point with a large degree is infected, it will have a greater infection ability and contagion capacity, so all the rumors about COVID-19 have higher peaks in the scale-free network. And, when μ_2_ decreases, it grows more in the scale-free network.

Figure 6 shows that in two network environments, before reaching the threshold, the density of the recovery increases when μ_2_ increases. No matter what kind of network it is, the densities of the recovery are the same when they reach the threshold. Therefore, even μ_2_ is controlled, the threshold of their stability cannot be lowered, but the density of the recovery during the diffusion of rumors about COVID-19 can still be changed and the efficiency of disproving rumors can be improved through corresponding the measures.

It can also be seen from Figure 7 that the increasement of the oyster density in the two networks when μ_2_ declines, indicating that when the variation's immune probability decreases, there are more variation in the network. Due to the silent mechanism, there will be more oyster. Therefore, the threshold of the oyster also increases when μ_2_ decrease.

Combined with Figures 5–7, in the entire network environment, when the variation's immune probability drops, fewer variation will change into the state of the recovery, the number of those who became the oyster will also increase, and the growth of those who turn into the recovery also increases more slowly and relatively. Because of the influence of the probability of the spreader turning into the variation, the reduction of the variation's immune probability does not significantly promote the diffusion of rumors about COVID-19, but it can observably increase the density of the recovery. For the government, strengthening the popularization of the truth to the variation can effectively promote the increasement of the recovery and of the widespread truth, which is notably conducive to the control the COVID-19 rumor.

### The Impact of the Variation's Silent Probability on Rumors About COVID-19 Diffusion

**Figure 8:**
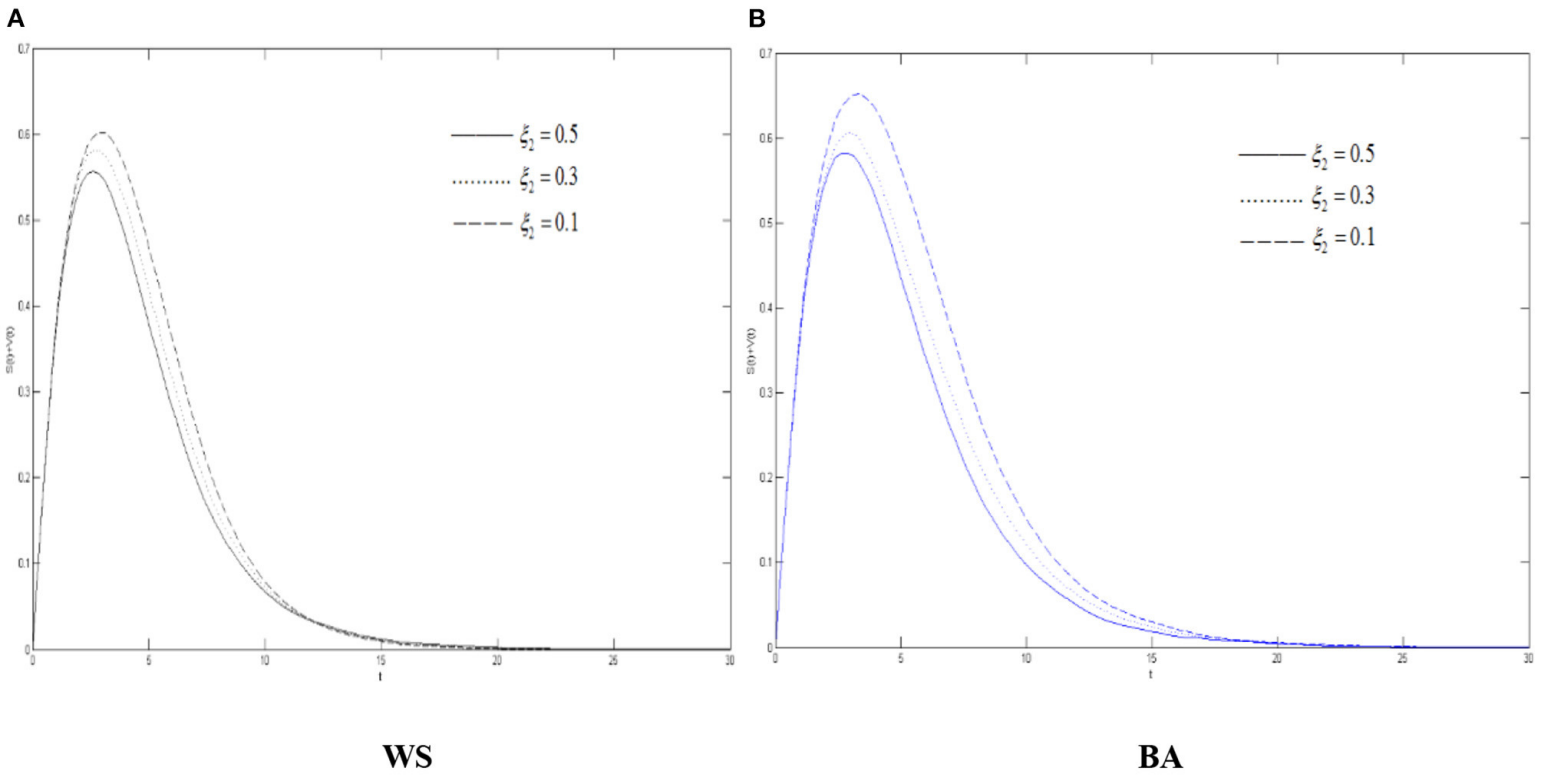
The differences of the diffuser density in WS small world and BA scale-free network when ξ_2_ changes. **(A)** WS, **(B)** BA.

It can be seen from Figure 8 that max{*S*(*t*) + *V*(*t*)} has experienced the process of continuous growth and falling after reaching the peak. With the increase of the variation's silent probability, max{*S*(*t*) + *V*(*t*)} declines. Meanwhile the COVID-19 rumor diffuses more slowly, indicating that in the process of diffusing the rumors about COVID-19, improving the ability of the community to calmly think about emergencies can well suppress the activation of the variation which could reduce the destructive effect of rumors about COVID-19. As seen from Figures 4, 7, compared with the variation's immune probability, the silent probability can increase the range and intensity of rumors about COVID-19 more greatly.

**Figure 9:**
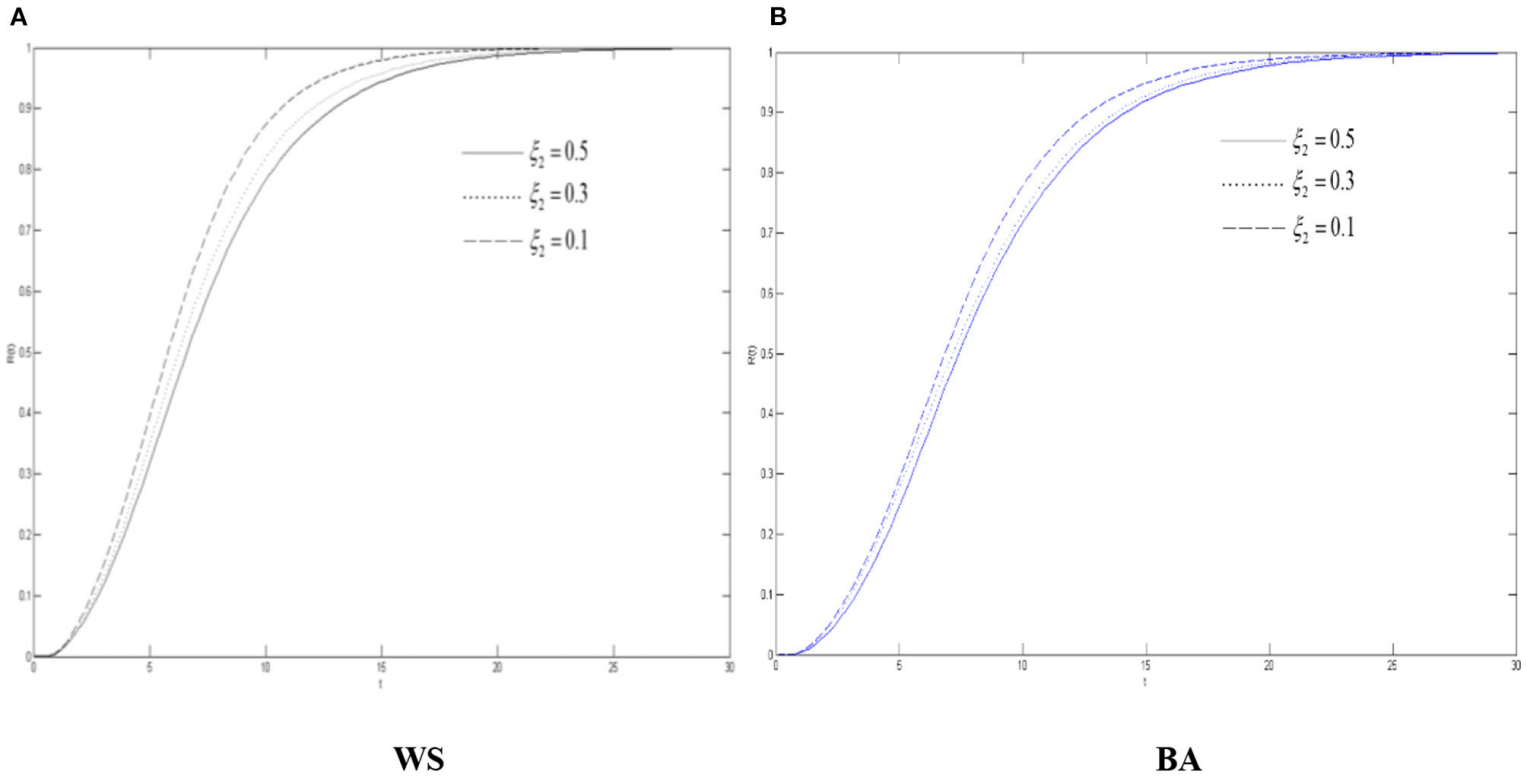
The differences of the recovery density in WS small world and BA scale-free network when ξ_2_ changes. **(A)** WS, **(B)** BA.

As can be seen from Figure 9, the density of the recovery *R*(*t*) in the two network environments has gone through a state of increasing and then becoming stable with time. As the variation's silent probability increases, the growth of *R*(*t*) becomes slower. Compared with the WS small-world network, it is growing more slowly in the BA scale-free network, which also shows that it is more difficult to refute rumors about COVID-19 in this environment. It can tell from Figure 9 that in both networks, both densities of the oyster *O*(*t*) and of the diffuser *S*(*t*) + *V*(*t*) have undergone a process of continuous growth and falling after reaching the peak. As ξ_2_ increases, the faster *O*(*t*) rises, the greater the corresponding peak value will be.

**Figure 10:**
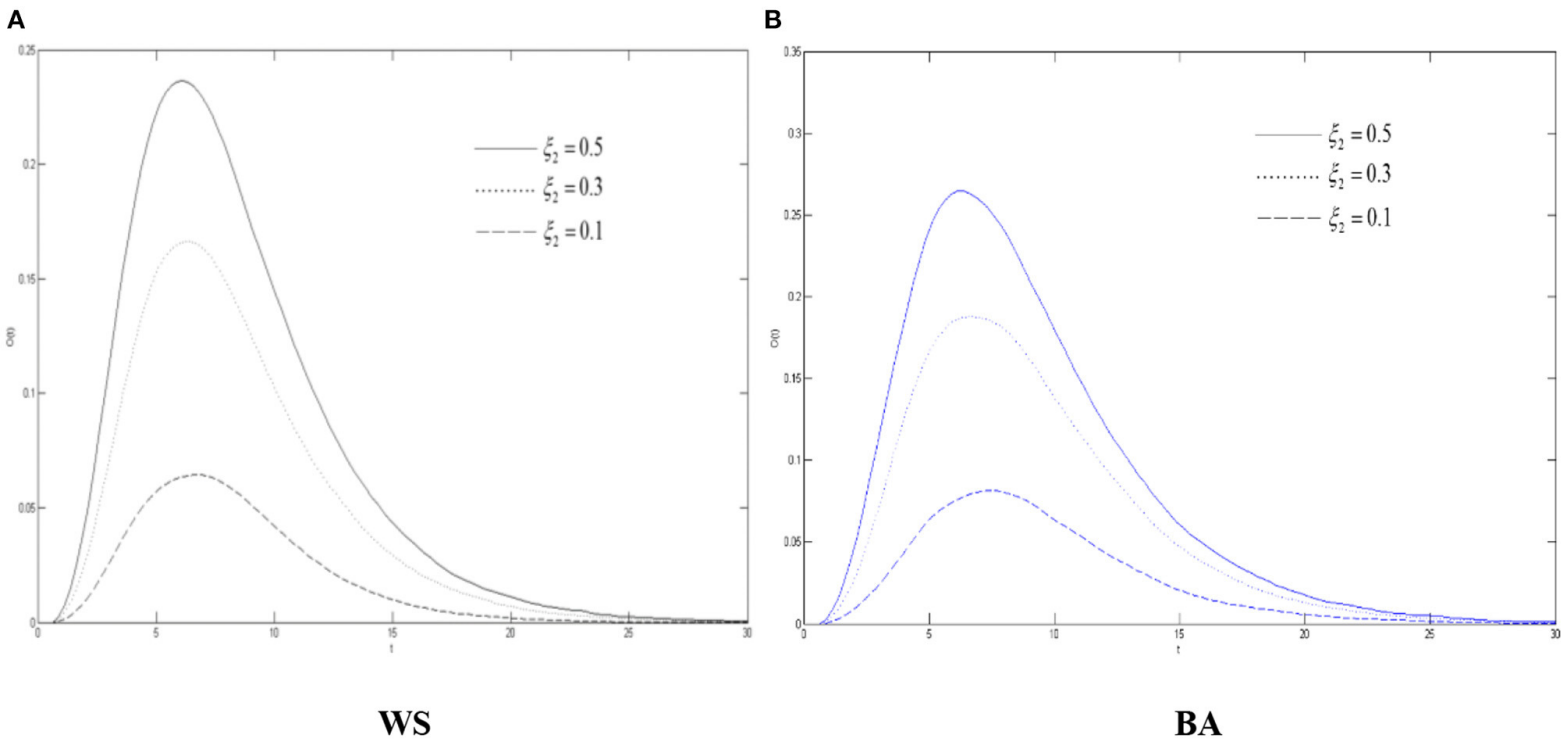
The differences of the oyster density in WS small world and BA scale-free network when ξ_2_ changes. **(A)** WS, **(B)** BA.

Combining Figures 8–10, it can be concluded that as variation's silent probability increases, more variation will become the oyster, and more diffuser in the network will tend to be “silent” and more functional as for the buffering effect of the COVID-19 rumor diffusion, more people in the group will calmly explore the truth of rumors about COVID-19, and there will be more truth carriers, thus, weakening the severity of rumors about COVID-19. For the same reason, the increasement of the silent probability of the spreaders also works to refute the rumors about COVID-19. Compared with Figures 5–7, it can be illustrated that if the variation's silent and immune probabilities both changed, the adjustment of the silent probability can control the diffusion of rumors about COVID-19 with a better result. As far as it is concerned from the conclusion when rumors about COVID-19 occur, people with a higher level of elaborative faculty will be more cautious about facing the unknown public opinion. The government should also improve the education of the relevant knowledge of the crowd and improve their identification ability.

### The Impact of the Spreader's Infected Probability on COVID-19 Rumor Diffusion

**Figure 11:**
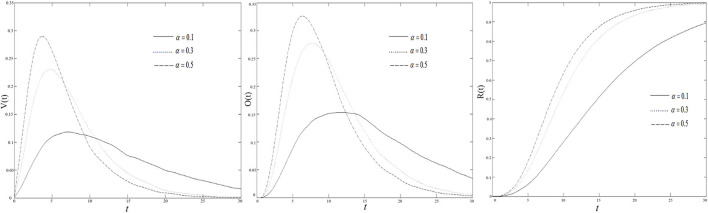
The density of variation, oyster, and recovery vary with alpha in BA scale-free network.

Figure 11 shows the changes of the density of the variation, the oyster, and the recovery in the BA scale-free network when the spreader's infected probabilities are 0.1, 0.3, and 0.5, respectively. As shown in Figure 11A, when the infected probability increases, the density of the variation has undergone a rapid increase to the threshold, and then decreased. And, when the infected probability is higher, the density of the variation increases faster, and the peak value that is reached is also larger. It can also be concluded from the changing curve in the figure that the initial rumors about COVID-19, with great infectivity, can quickly affect the surrounding people when diffusing in the group, making more and more groups infected with rumors about COVID-19, which leads to a rapid increase of the variation. But, in the middle and late stages of the COVID-19 rumor propagation, the growth of the infected probability from the figure will cause the amount of the variation to fall faster after reaching the threshold, and it cannot keep the variation in a state of a large quantity. Therefore, it can be concluded that if there are more information communicators in the social group, diffusing the information versions and contents tend to be more consistent, which proves that the more sufficient each individual information is, the faster the quantity of people who spread rumors about COVID-19 drops, the less rumors about COVID-19 are likely to diffuse.

From Figure 11B when the infected probability increases, the density of the oyster also undergoes a process of swiftly increasing to the threshold, and then decreasing. When the infected probability is higher, the oyster density goes up faster, and the threshold reached is also larger. Combining Figures 11A,B, the rise of infected probability will lead to more variation and more oyster. In the middle and late stages of the COVID-19 rumor diffusion, as the quantity of all diffusers increased, the COVID-19 rumor content or version begins to stabilize, and the quantity of variation grows slowly or even decreases, but the number of the oyster did not increase accordingly. With the changes in the scale-free network of density of the recovery in Figure 11C, the reduced variation does not become the oyster, and, on the contrary, turn to the state of the recovery. It can be concluded that when the infected probability increases, the thresholds of the variation and the oyster will increase, but with its large quantity, it is difficult to maintain stability. Everyone will have multiple versions of the content at first, but when there is more information about COVID-19 communicators in the social group, the version or content will quickly tend to be unanimous. The more accurate the information everyone understands, the less likely it is to diffuse rumors about COVID-19.

## Conclusion

In view of the complex rumors about the COVID-19, we modified the traditional SIR model, and a new rumor propagation model incorporating “variation” and “oyster” is proposed. At the same time, influences of the variation and the oyster in the diffusion of rumors about COVID-19 are demonstrated, and are placed into the WS small world network and the BA scale free network for comparison. The increase of the immune and silence probability of the variation can effectively improve and reduce the density of diffusion of the propagation of truth in the group. A higher infected probability will bring more people who diffuse the rumors about COVID-19, but it will not always maintain a large quantity of the variation. The more information disseminators in the society, the more easily the version and content of the disseminated information tend to be consistent. It can prove that with more adequate information among individuals, the amount of people who diffuse the rumors about COVID-19 drops more quickly, and the chance of diffusing rumors about COVID-19 could be smaller.

First, at the beginning of the rumors about COVID-19, the information must be disclosed in a timely and effective manner. For the government, strengthening the promotion of the truth to the variation and the spreader, rather than blocking the message, can effectively increase the quantity of the recovery and the widespread dissemination of truth, which are significative and conducive to the control of rumors about COVID-19. Secondly, the oysters, which are silent and often possesses good skepticism ability, thus, availably guide the people who are captivated by the rumors about COVID-19, which will play a positive role in shaping the public opinion about COVID-19. Thirdly, compared with non-response, timely and effective interpretation can improve the ability of the group to distinguish rumors about COVID-19. In the meantime, it provides a new model for the related departments to solve the phenomenon of increasingly widespread internet COVID-19 rumors, which has certain practical guiding significance. This study about the individual's own situation is not detailed enough. The next stage will study the interaction between individuals at the micro level in the context of the spread of epidemic rumors. In the future, the research results can be applied into various fields including personalized recommendation (33–35), anomaly detection (36, 37), sustainable tourism (38, 39), personal health (40), and so on.

## Data Availability Statement

The original contributions presented in the study are included in the article/supplementary material, further inquiries can be directed to the corresponding author.

## Author Contributions

CJ designed the study and conceived the manuscript. FB and CX implemented the simulation experiments. YJ, FB, and BZ drafted the manuscript. CX and BZ were involved in revising the manuscript. All authors were involved in writing the manuscript and approve of its final version.

## Funding

This research was funded by Natural Science Foundation of Zhejiang Province (Nos. LQ20G010002 and LY20G010001), the National Science Foundation of China (No. 71702164), the project of China (Hangzhou) Cross-border E-commerce College (No. 2021KXYJ06), the Philosophy and Social Science Foundation of Zhejiang Province (No. 21NDJC083YB), and Contemporary Business and Trade Research Center of Zhejiang Gongshang University (Nos. XT202103 and XT202105).

## Conflict of Interest

The authors declare that the research was conducted in the absence of any commercial or financial relationships that could be construed as a potential conflict of interest.

## Publisher's Note

All claims expressed in this article are solely those of the authors and do not necessarily represent those of their affiliated organizations, or those of the publisher, the editors and the reviewers. Any product that may be evaluated in this article, or claim that may be made by its manufacturer, is not guaranteed or endorsed by the publisher.
